# Interventional Procedures in Deep Venous Thrombosis Treatment: A Review of Techniques, Outcomes, and Patient Selection

**DOI:** 10.3390/medicina61081476

**Published:** 2025-08-18

**Authors:** Arkadiusz Kacała, Mateusz Dorochowicz, Jędrzej Fischer, Adrian Korbecki, Aleksander Truszyński, Anna Madura, Krzysztof Dyś, Andrzej Szuba, Maciej Guziński

**Affiliations:** 1 Department of General, Interventional and Neuroradiology, Wroclaw Medical University, 50-367 Wrocław, Poland; 2 Department of General, Interventional and Neuroradiology, Wroclaw University Hospital, 50-556 Wrocław, Poland; 3 Department and Clinic of Angiology and Internal Medicine, Wroclaw Medical University, 50-367 Wrocław, Poland

**Keywords:** deep venous thrombosis, catheter-directed thrombolysis, mechanical thrombectomy, pharmacomechanical thrombectomy, venous stenting, post-thrombotic syndrome

## Abstract

*Background and Objectives:* Deep venous thrombosis (DVT) is associated with pulmonary embolism and long-term complications such as post-thrombotic syndrome (PTS). Anticoagulation prevents thrombus extension but does not actively remove clot. Interventional techniques, including catheter-directed thrombolysis, mechanical and pharmacomechanical thrombectomy, and venous stenting, have been introduced to restore venous patency and reduce complications. This systematic review summarizes current evidence on outcomes, safety, and patient selection for these procedures. *Materials and Methods:* A systematic search of PubMed, EMBASE, Cochrane Library, and Web of Science was conducted for studies published between January 2000 and February 2024. Eligible studies included randomized controlled trials, systematic reviews, meta-analyses, and observational studies with ≥20 patients. Extracted outcomes were technical success, thrombus clearance, venous patency, PTS, quality of life, and complications. Risk of bias was assessed using the Cochrane Risk of Bias Tool, Newcastle–Ottawa Scale, and AMSTAR-2. *Results:* Of 456 records screened, 35 studies were included. Randomized trials (CaVenT, ATTRACT, CAVA) showed that catheter-directed and pharmacomechanical approaches improved venous patency and reduced moderate-to-severe PTS in selected patients with iliofemoral DVT, though overall benefit was variable. Mechanical thrombectomy devices (e.g., AngioJet, ClotTriever, FlowTriever) achieved high thrombus clearance and shorter procedural times, with device-specific complication profiles. Observational data demonstrated venous stenting patency rates of 74–89% at 12 months. Study heterogeneity limited direct comparisons. *Conclusions:* Interventional procedures can reduce PTS and improve outcomes in carefully selected patients, particularly those with acute iliofemoral DVT. Modern mechanical and pharmacomechanical techniques enhance efficiency and safety, while venous stenting addresses underlying obstructions. Further high-quality trials with long-term follow-up are needed to define optimal patient selection and comparative effectiveness.

## 1. Introduction

Deep venous thrombosis (DVT) represents a significant global health burden, affecting approximately 1–2 individuals per 1000 annually in the general population [1,2]. This condition is characterized by the formation of blood clots within the deep veins, most commonly in the lower extremities, and carries substantial morbidity and mortality risks. The clinical significance of DVT extends beyond its acute presentation, as it can lead to life-threatening complications such as pulmonary embolism (PE) and debilitating long-term sequelae including post-thrombotic syndrome (PTS) [3].

The pathophysiology of DVT is often explained by Virchow’s triad: venous stasis, endothelial injury, and hypercoagulability [4]. Various risk factors contribute to the development of DVT, including prolonged immobilization, surgery, trauma, malignancy, pregnancy, hormonal therapy, and inherited or acquired thrombophilias [5]. The anatomical location of thrombosis significantly influences both the acute management approach and long-term outcomes. Proximal DVT, particularly iliofemoral DVT (IFDVT), carries a worse prognosis compared to distal DVT, with higher rates of PTS and recurrent thromboembolism [6,7].

Post-thrombotic syndrome, characterized by chronic pain, edema, skin changes, and in severe cases, venous ulceration, affects up to 50% of patients within two years following symptomatic lower extremity DVT [8,9]. This chronic complication substantially impairs quality of life and imposes significant healthcare costs. Patients with severe PTS have quality-of-life metrics comparable to those with chronic conditions such as cancer or congestive heart failure [10]. The economic burden of DVT and its complications is substantial, with annual costs estimated in the billions of dollars in developed countries [11].

Historically, the management of DVT has centered on anticoagulation therapy, which effectively prevents thrombus propagation and reduces the risk of pulmonary embolism. However, anticoagulation alone does not actively remove existing thrombus, leaving patients vulnerable to the development of PTS due to residual venous obstruction and valvular damage [12]. This limitation has driven the development and refinement of interventional approaches aimed at actively removing thrombus and restoring venous patency.

The evolution of interventional techniques for DVT treatment has progressed significantly over the past few decades. Early approaches included systemic thrombolysis and surgical thrombectomy, which, while effective in some cases, were associated with high rates of bleeding complications and procedural morbidity [13,14]. The advent of catheter-directed techniques in the 1990s marked a paradigm shift, allowing for targeted thrombolytic delivery with reduced systemic exposure [15]. Subsequently, mechanical and pharmacomechanical thrombectomy devices have further expanded the interventional armamentarium, offering options for more rapid thrombus removal with potentially lower bleeding risks [16].

Recent guidelines from major cardiovascular and interventional societies have increasingly recognized the role of interventional approaches in selected DVT patients [17,18]. The American Heart Association, the Society of Interventional Radiology, and the European Society for Vascular Surgery have all published recommendations addressing the use of catheter-directed therapies for specific DVT populations, particularly those with extensive iliofemoral involvement, severe symptoms, or low bleeding risk [19,20,21].

Despite these advances, considerable debate persists regarding the optimal selection of patients for interventional treatment, the timing of intervention, the choice of specific techniques, and the long-term benefits relative to risks and costs [22]. Several randomized controlled trials and large observational studies have attempted to address these questions, with varying results [18,23,24]. The heterogeneity in study designs, patient populations, interventional techniques, and outcome measures has contributed to the ongoing uncertainty in this field.

This review aims to synthesize the current evidence regarding interventional procedures for DVT treatment, with a focus on catheter-directed thrombolysis, mechanical thrombectomy, pharmacomechanical thrombectomy, and venous stenting. We examine the technical aspects, efficacy outcomes, safety profiles, and patient selection considerations for each approach. Additionally, we explore emerging technologies, ongoing clinical trials, and areas of uncertainty that warrant further investigation. By providing an evidence-based overview of the current state of interventional DVT management, this review seeks to inform clinical decision-making and highlight future research directions in this rapidly evolving field.

## 2. Methods

### 2.1. Search Strategy and Study Selection

This review was conducted in accordance with the Preferred Reporting Items for Systematic Reviews and Meta-Analyses (PRISMA) guidelines. A comprehensive literature search was performed using PubMed, EMBASE, Cochrane Library, and Web of Science databases for articles published between January 2000 and February 2024. The search strategy included combinations of Medical Subject Headings (MeSH) terms and keywords related to deep venous thrombosis and interventional procedures: “deep vein thrombosis,” “deep venous thrombosis,” “DVT,” “iliofemoral DVT,” “catheter-directed thrombolysis,” “mechanical thrombectomy,” “pharmacomechanical thrombectomy,” “venous stenting,” “endovascular therapy,” “interventional treatment,” and “post-thrombotic syndrome.”

**Figure 1:**
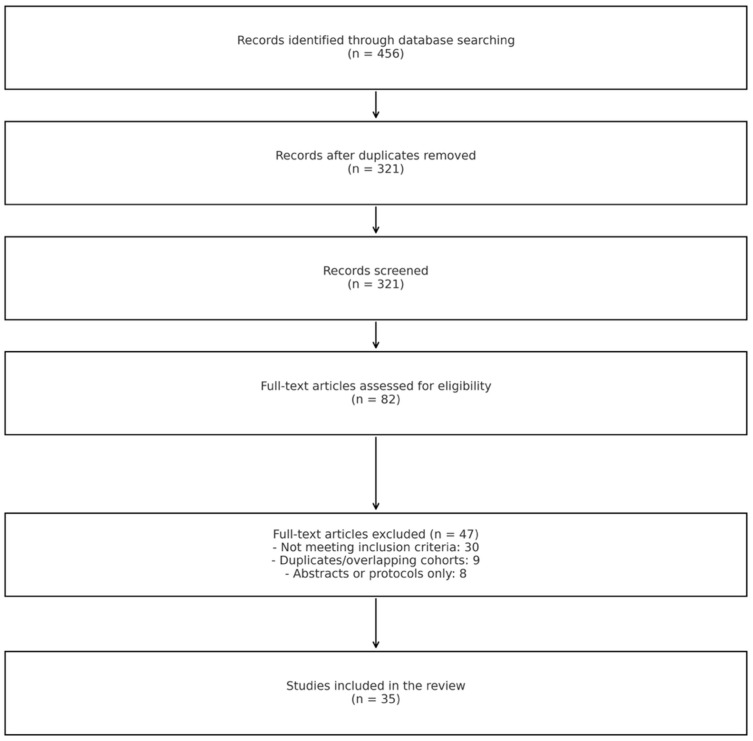
PRISMA 2020 flow diagram illustrating the study selection process. A total of 456 records were identified through database searching. After removing duplicates, 321 records were screened by title and abstract. Of these, 82 full-text articles were assessed for eligibility, and 35 studies were ultimately included in the review. Reasons for exclusion at the full-text stage are detailed in the diagram.

Inclusion criteria were as follows: (1) randomized controlled trials, systematic reviews, meta-analyses, or observational studies with at least 20 patients; (2) studies evaluating catheter-directed thrombolysis, mechanical thrombectomy, pharmacomechanical thrombectomy, or venous stenting for DVT; (3) studies reporting at least one of the following outcomes: technical success, thrombus removal, venous patency, post-thrombotic syndrome, quality of life, or complications; and (4) full-text articles in English. Exclusion criteria were as follows: (1) case reports, letters, editorials, or conference abstracts; (2) studies focusing exclusively on pulmonary embolism without DVT; (3) studies evaluating only systemic thrombolysis or surgical thrombectomy; and (4) animal or in vitro studies. The study selection process is summarized in the PRISMA flow diagram (Figure 1).

Two independent reviewers screened titles and abstracts for eligibility, followed by full-text review of potentially relevant articles. Disagreements were resolved by consensus or by a third reviewer. Reference lists of included studies and relevant review articles were manually searched for additional eligible studies.

A completed PRISMA checklist is included in the Supplementary Materials. This review was not prospectively registered in a review database such as PROSPERO. A study protocol was not published prior to conducting the review.

### 2.2. Data Extraction and Quality Assessment

Data extraction was performed independently by two reviewers using a standardized form. The following information was collected: study characteristics (design, year, country), patient demographics (age, sex, DVT location, symptom duration), intervention details (technique, devices, protocols), outcome measures (technical success, thrombus removal, venous patency, post-thrombotic syndrome, quality of life, complications), and follow-up duration.

The methodological quality of randomized controlled trials was assessed using the Cochrane Risk of Bias tool, evaluating random sequence generation, allocation concealment, blinding, incomplete outcome data, selective reporting, and other sources of bias. For observational studies, the Newcastle–Ottawa Scale was used, evaluating selection of study groups, comparability of groups, and ascertainment of exposure or outcome. The quality of systematic reviews and meta-analyses was assessed using the AMSTAR-2 (A MeaSurement Tool to Assess systematic Reviews) instrument.

While individual studies were assessed using appropriate tools (Cochrane Risk of Bias tool, Newcastle–Ottawa Scale, AMSTAR-2), a pooled summary of risk of bias was not compiled due to heterogeneity in study designs. Most included randomized controlled trials demonstrated moderate risk due to lack of blinding, while observational studies showed variable risk depending on outcome reporting and follow-up duration.

The certainty of evidence was not formally rated using GRADE or a similar tool. However, inferences were drawn based on study design hierarchy, sample size, methodological quality, and consistency of results across studies. Future analyses should consider applying formal evidence grading frameworks.

### 2.3. Outcome Measures

The primary outcomes of interest were (1) technical success, defined as successful catheter placement and completion of the intended procedure; (2) degree of thrombus removal, categorized as complete (>90% removal), partial (50–90% removal), or minimal (<50% removal); (3) incidence of post-thrombotic syndrome, preferably assessed using validated tools such as the Villalta scale or the Venous Clinical Severity Score; and (4) major bleeding complications, defined according to the International Society on Thrombosis and Haemostasis criteria.

Secondary outcomes included (1) venous patency rates (primary, primary-assisted, and secondary) at various time points; (2) quality-of-life measures; (3) procedural time and hospital length of stay; (4) minor bleeding complications; (5) other procedure-related complications; and (6) cost-effectiveness metrics when available.

### 2.4. Data Synthesis and Analysis

Due to the anticipated heterogeneity in study designs, interventions, and outcome measures, a narrative synthesis approach was planned rather than a formal meta-analysis. Studies were grouped by intervention type (catheter-directed thrombolysis, mechanical thrombectomy, pharmacomechanical thrombectomy, and venous stenting) and by study design (randomized controlled trials versus observational studies). When multiple studies reported on the same outcome using similar definitions, ranges and weighted averages were calculated. Results were presented with consideration of study quality, sample size, and follow-up duration.

Due to the heterogeneity of included studies and lack of quantitative meta-analysis, a formal assessment of reporting bias (e.g., funnel plots or statistical tests) was not performed. Potential publication bias was qualitatively considered during data synthesis by evaluating study design, sample size, and consistency across findings.

## 3. Conventional Treatment Approaches

The management of deep venous thrombosis (DVT) has evolved significantly over the past several decades, with conventional treatment approaches forming the foundation upon which more advanced interventional strategies have been developed. This section examines the standard therapeutic options that have historically been employed for DVT management, their mechanisms of action, efficacy, limitations, and the evidence supporting their use.

### 3.1. Anticoagulation Therapy

Anticoagulation remains the cornerstone of DVT treatment, with a primary goal of preventing thrombus propagation, reducing the risk of pulmonary embolism, and allowing for natural fibrinolytic processes to gradually dissolve the existing clot [25,26]. Several classes of anticoagulants are currently employed in clinical practice, each with distinct pharmacological properties, administration routes, monitoring requirements, and safety profiles.

#### 3.1.1. Unfractionated Heparin

Unfractionated heparin (UFH) was one of the earliest anticoagulants used for DVT treatment and continues to play an important role in specific clinical scenarios. UFH acts by binding to antithrombin, enhancing its inhibitory effect on thrombin and factor Xa [27]. The recommended initial anticoagulation dosage for patients presenting with DVT is an intravenous bolus of 80 units/kg followed by a continuous infusion of 18 units/kg/hr, with dose adjustments to target an activated partial thromboplastin time (aPTT) of 1.5–2.5 times the control value or plasma heparin levels of 0.3–0.7 IU/mL anti-Xa activity [28,29].

The advantages of UFH include its short half-life, reversibility with protamine sulfate, and ability to be used in patients with severe renal impairment [30,31]. However, UFH therapy requires frequent laboratory monitoring, carries a risk of heparin-induced thrombocytopenia (HIT), and necessitates continuous intravenous administration, limiting its outpatient use [32]. Despite these limitations, UFH remains valuable for hospitalized patients, particularly those with renal failure, high bleeding risk, or potential need for urgent procedures or surgery.

#### 3.1.2. Low Molecular Weight Heparin

Low molecular weight heparins (LMWHs), derived from the depolymerization of UFH, have largely supplanted UFH as the preferred initial anticoagulant for most patients with DVT [33]. LMWHs primarily inhibit factor Xa with less effect on thrombin compared to UFH, resulting in a more predictable anticoagulant response [34]. Common LMWH preparations include enoxaparin (1 mg/kg twice daily or 1.5 mg/kg once daily), dalteparin (200 IU/kg once daily), and tinzaparin (175 anti-Xa IU/kg once daily) [35,36].

The advantages of LMWHs include subcutaneous administration, predictable dose-response relationship, minimal need for laboratory monitoring in most patients, lower risk of HIT, and the possibility of outpatient treatment [37]. Multiple randomized controlled trials have demonstrated that LMWHs are at least as effective as UFH for preventing recurrent venous thromboembolism (VTE), with a similar or lower risk of bleeding complications and reduced mortality [38,39]. These benefits, coupled with the convenience of once- or twice-daily subcutaneous injections, have established LMWHs as the standard of care for initial DVT treatment in most clinical scenarios [40].

#### 3.1.3. Direct Oral Anticoagulants

Direct oral anticoagulants (DOACs) represent the most recent advancement in anticoagulation therapy for DVT. This class includes direct thrombin inhibitors (dabigatran) and factor Xa inhibitors (rivaroxaban, apixaban, edoxaban) [41]. DOACs offer several advantages over traditional anticoagulants, including oral administration, fixed dosing without routine laboratory monitoring, rapid onset of action, and fewer drug and food interactions compared to vitamin K antagonists [42]. Multiple large randomized controlled trials have demonstrated that DOACs are non-inferior to conventional therapy (LMWH followed by vitamin K antagonists) for the treatment of acute DVT, with similar efficacy in preventing recurrent VTE and a generally favorable safety profile [43,44,45]. A meta-analysis of these trials showed that DOACs were associated with a 39% relative risk reduction in major bleeding compared to conventional therapy [46]. Based on this evidence, current guidelines from the American College of Chest Physicians (ACCP) and other major societies recommend DOACs over vitamin K antagonists for the treatment of DVT in patients without cancer, severe renal impairment, or other contraindications [47,48].

#### 3.1.4. Vitamin K Antagonists

Vitamin K antagonists (VKAs), primarily warfarin, were the mainstay of long-term anticoagulation for DVT before the advent of DOACs. VKAs inhibit the synthesis of vitamin K-dependent clotting factors (II, VII, IX, and X) and anticoagulant proteins C and S [49]. Warfarin therapy typically begins with an initial dose of 5–10 mg daily, with subsequent dose adjustments to achieve an international normalized ratio (INR) of 2.0–3.0 [49].

The limitations of VKAs include a narrow therapeutic window, numerous drug and food interactions, genetic variability in metabolism, and the need for regular INR monitoring and dose adjustments [47]. Despite these challenges, VKAs remain an important option for patients with contraindications to DOACs, such as those with mechanical heart valves, antiphospholipid syndrome, or severe renal impairment [50].

### 3.2. Systemic Thrombolysis

Systemic thrombolysis involves the intravenous administration of fibrinolytic agents to accelerate clot dissolution. Agents used for this purpose include streptokinase, urokinase, and recombinant tissue plasminogen activator (rt-PA) [51]. These medications activate plasminogen to form plasmin, which degrades fibrin and dissolves thrombi [52].

Early studies demonstrated that systemic thrombolysis could achieve more rapid and complete clot lysis compared to anticoagulation alone [53]. However, this approach is associated with a significantly increased risk of major bleeding, including intracranial hemorrhage, with rates ranging from 6% to 44% across various trials [3,37]. A systematic review by Watson et al. found that while systemic thrombolysis reduced the risk of post-thrombotic syndrome (RR 0.66, 95% CI 0.47–0.94), this benefit came at the cost of a more than twofold increase in bleeding complications (RR 2.23, 95% CI 1.41–3.52) [54].

Due to this unfavorable risk-benefit profile, current guidelines generally do not recommend systemic thrombolysis for routine DVT treatment [55]. Its use is typically reserved for patients with massive iliofemoral DVT causing limb-threatening ischemia (phlegmasia cerulea dolens) when more targeted interventional approaches are not available or feasible [56].

### 3.3. Compression Therapy

Compression therapy, using graduated compression stockings (GCS) or bandages, has historically been recommended as an adjunctive treatment for DVT to reduce acute symptoms and prevent post-thrombotic syndrome [18]. The proposed mechanisms include reduction of venous diameter, increased venous flow velocity, improved venous valve function, and enhanced fibrinolysis [57].

Early observational studies and small randomized trials suggested that compression therapy could significantly reduce the incidence of PTS [21,58]. However, the landmark SOX trial, a large multicenter randomized controlled trial involving 806 patients with proximal DVT, found no benefit of elastic compression stockings (30–40 mmHg) compared to placebo stockings in preventing PTS over a 2-year follow-up period (hazard ratio 1.13, 95% CI 0.88–1.44) [16]. These findings led to a downgrading of recommendations for routine use of compression stockings in recent guidelines [59].

Nevertheless, compression therapy remains valuable for symptom management in patients with acute DVT and established PTS [60]. The optimal compression modality, pressure, and duration remain subjects of ongoing research, with some evidence suggesting that higher pressures (>30 mmHg) and better compliance may be associated with improved outcomes [52,61].

### 3.4. Limitations of Conventional Approaches

While anticoagulation effectively prevents thrombus propagation and reduces the risk of pulmonary embolism, it has several important limitations in the management of DVT. First, anticoagulation does not actively remove existing thrombus but relies on the body’s natural fibrinolytic mechanisms, which may be slow and incomplete, particularly for extensive clots [62]. Studies using serial venographic assessment have shown that complete lysis occurs in only 10–15% of patients treated with anticoagulation alone, with 50–80% developing some degree of residual venous obstruction [63,64].

Second, delayed or incomplete thrombus resolution can lead to venous valve damage and chronic venous insufficiency, contributing to the development of PTS [65]. Despite optimal anticoagulation therapy, PTS develops in 20–50% of patients following proximal DVT, with severe manifestations occurring in 5–10% [66]. The risk is particularly high in patients with iliofemoral DVT, with PTS rates exceeding 50% in some studies [3].

Third, anticoagulation carries an inherent risk of bleeding complications, which must be balanced against its therapeutic benefits. Major bleeding occurs in approximately 2–3% of patients during the initial 3 months of anticoagulation, with rates varying based on patient characteristics, anticoagulant choice, and treatment intensity [7,37]. This risk limits the use of anticoagulation in patients with active bleeding or high bleeding risk.

These limitations of conventional therapy, particularly for patients with extensive proximal DVT, have driven interest in interventional approaches that can actively remove thrombus, potentially reducing the risk of PTS while maintaining an acceptable safety profile. The following sections will examine these interventional strategies in detail.

## 4. Interventional Procedures for DVT

Interventional procedures for deep venous thrombosis have evolved significantly over the past three decades, offering options for active thrombus removal and restoration of venous patency. This section examines the major interventional approaches currently employed in clinical practice, including their technical aspects, procedural considerations, and device-specific characteristics.

### 4.1. Catheter-Directed Thrombolysis

**Figure 2:**
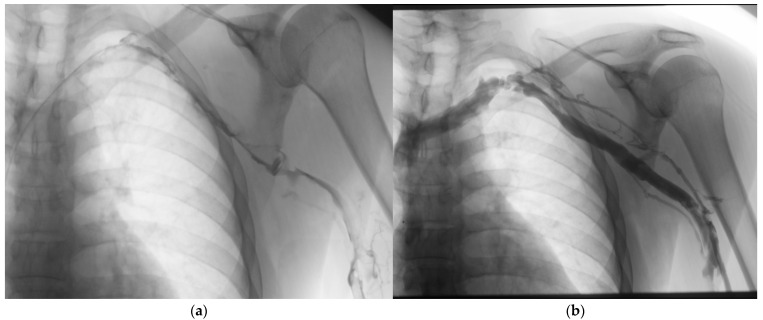
Venogram showing a thrombolysis catheter in place. (**a**) Deep vein thrombosis (DVT) of the left upper extremity in the acute phase, with extensive thrombus visible in the left subclavian, axillary, and brachial veins. (**b**) Follow-up image the next day after catheter-directed thrombolysis, demonstrating effective resolution of the thrombus. A residual stenosis is visible at the typical site between the first rib and clavicle.

Catheter-directed thrombolysis (CDT) represents one of the earliest and most extensively studied interventional approaches for DVT treatment. This technique involves the direct delivery of fibrinolytic agents into the thrombus via an endovascular catheter, allowing for targeted therapy with lower systemic exposure compared to intravenous thrombolysis [15] (Figure 2).

#### 4.1.1. Technical Aspects

The procedure typically begins with venous access, most commonly via the popliteal or common femoral vein, guided by ultrasound [19]. For iliofemoral DVT, a contralateral approach may be preferred to avoid traversing the thrombus during initial access [67]. After venography confirms the extent of thrombosis, a multi-sidehole infusion catheter or wire is positioned within the thrombus [52]. Some operators employ the “crossing catheter technique,” where the catheter is advanced through the entire thrombus, while others use the “lacing technique,” involving multiple passes through different portions of the clot [68].

The most commonly used thrombolytic agents include recombinant tissue plasminogen activator (rt-PA, alteplase), reteplase, and urokinase [69]. Typical dosing regimens for rt-PA range from 0.5 to 1.0 mg/hour, with total doses generally not exceeding 24–48 mg [62]. The infusion is continued for 24–72 h, with interval venography performed every 8–24 h to assess progress and reposition the catheter as needed [16]. Concomitant anticoagulation with unfractionated heparin is typically administered at subtherapeutic doses (500–800 units/hour) to prevent pericatheter thrombosis [19].

#### 4.1.2. Adjunctive Techniques

Several adjunctive techniques have been developed to enhance the efficacy of CDT. Pulse-spray thrombolysis involves the forceful injection of small boluses of thrombolytic agent to mechanically disrupt the clot and increase drug–thrombus contact [70]. Ultrasound-assisted thrombolysis employs catheters that generate high-frequency, low-energy ultrasound waves to disaggregate fibrin strands and enhance drug penetration [71]. The EKOS EndoWave system (Boston Scientific, Marlborough, MA, USA) is the most widely studied device in this category, with the BERNUTIFUL trial demonstrating reduced treatment time compared to standard CDT (13.7 vs. 21.4 h, *p* = 0.004) [66].

Balloon maceration involves the use of angioplasty balloons to physically disrupt the thrombus and create channels for improved drug delivery [72]. This technique is particularly useful for chronic or organized thrombi that may be resistant to thrombolysis alone [73]. Following successful thrombolysis, balloon angioplasty and/or stenting may be performed to address underlying venous stenoses or obstructions, which are present in up to 80% of patients with iliofemoral DVT [74].

### 4.2. Mechanical Thrombectomy

Mechanical thrombectomy (MT) encompasses a variety of techniques and devices designed to physically remove thrombus without primary reliance on pharmacological thrombolysis. These approaches offer potential advantages of more rapid thrombus clearance, reduced or eliminated need for thrombolytic agents, and shorter procedure times [16].

#### 4.2.1. Device Categories and Mechanisms

**Figure 3:**
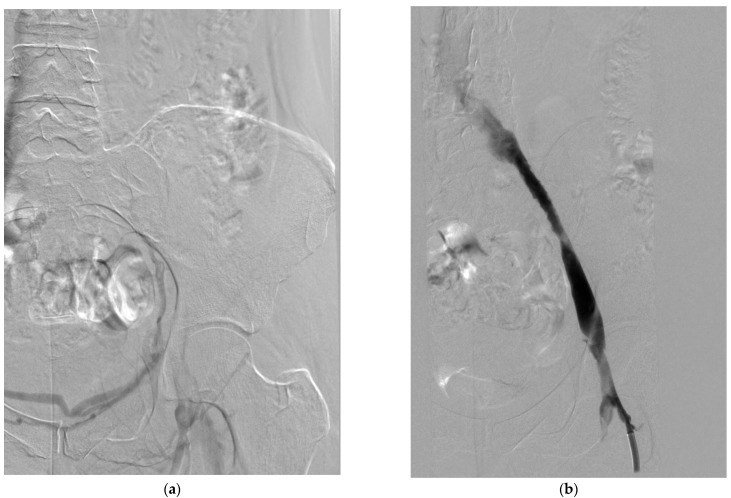
Venogram demonstrating aspiration treatment in deep vein thrombosis (DVT). (**a**) Acute-phase DVT of the left lower extremity, with extensive thrombus causing occlusion of the common and external iliac veins. (**b**) Post-aspiration thrombectomy venogram showing a reduced thrombus burden and patent iliac veins, with residual stenosis in the typical location suggestive of May–Thurner syndrome.

Mechanical thrombectomy devices can be broadly categorized based on their mechanism of action:

1. Rheolytic thrombectomy: These systems use high-velocity saline jets to create a Venturi effect, fragmenting and aspirating thrombus. The AngioJet system (Boston Scientific, Marlborough, MA, USA) is the most widely used device in this category [75]. It employs a dual-lumen catheter with high-pressure jets that create a localized low-pressure zone, drawing thrombus into the catheter for fragmentation and removal [76]. The system can be used in “thrombectomy mode” for pure mechanical removal or in “power pulse mode” for combined pharmacomechanical therapy [70].
2. Rotational thrombectomy: These devices employ rotating components to macerate thrombus. The Aspirex device (Straub Medical, Wangs, Switzerland) uses a rotating helix within a catheter to fragment and aspirate thrombus [77]. Cleaner Rotational Thrombectomy System (Argon Medical Devices, Plano, TX, USA) features a sinusoidal wire that rotates at high speed (3500 rpm) to macerate thrombus without aspiration [65].
3. Aspiration thrombectomy: These systems rely on negative pressure to remove thrombus. The Indigo/Lightening System (Penumbra, Alameda, CA, USA) uses a vacuum pump to generate continuous suction through specially designed catheters [67]. The ClotTriever system (Inari Medical, Irvine, CA, USA) employs a mechanical retriever with a nitinol coring element and attached collection bag to engage and remove thrombus en bloc [78] (Figure 3).
4. Balloon-based systems: The FlowTriever system (Inari Medical, Irvine, CA, USA) uses large self-expanding nitinol disks to engage and extract thrombus, with an option for aspiration through the guide catheter [79]. This system is designed for large-vessel thrombectomy without the need for thrombolytics [79].

#### 4.2.2. Procedural Considerations

Mechanical thrombectomy procedures typically begin with venous access similar to CDT, followed by venography to assess thrombus extent [19]. Device selection depends on thrombus characteristics, vessel size, and operator preference [67]. Most procedures are performed under conscious sedation, though general anesthesia may be preferred for complex cases or prolonged procedures [16].

Technical limitations include difficulty navigating tortuous vessels, potential for vessel injury with aggressive manipulation, and incomplete thrombus removal, particularly with organized or wall-adherent thrombi [73]. Device-specific complications include hemolysis and bradyarrhythmias with rheolytic thrombectomy, necessitating caution when treating large thrombus volumes [80].

### 4.3. Pharmacomechanical Thrombectomy

Pharmacomechanical thrombectomy (PMT) combines mechanical disruption with localized thrombolytic delivery, aiming to enhance efficacy while minimizing thrombolytic dose and treatment duration [16]. This hybrid approach has gained popularity due to its potential to address the limitations of both CDT (long infusion times, higher bleeding risk) and pure mechanical thrombectomy (incomplete removal of organized thrombus) [67].

#### 4.3.1. Techniques and Devices

Several PMT techniques have been described:

1. Isolated thrombolysis: The Trellis system (Medtronic, now discontinued) isolated the treatment segment between two balloons while a rotating wire fragmented the thrombus and distributed the thrombolytic agent [81]. After a short dwell time (typically 15–20 min), the liquefied thrombus was aspirated before balloon deflation [82].
2. Power pulse delivery: The AngioJet system can be used in “power pulse mode,” where thrombolytic agent is forcefully injected into the thrombus, allowed to dwell for 20–30 min, and then removed using the standard thrombectomy mode [70]. This technique has been shown to reduce procedure time and thrombolytic dose compared to standard CDT [76].
3. Percutaneous mechanical thrombectomy with thrombolysis: This approach involves initial mechanical thrombectomy followed by a short-duration thrombolytic infusion (typically 4–6 h) to address residual thrombus [72]. This sequential approach may be particularly useful for extensive or partially organized thrombi [73].

#### 4.3.2. Procedural Outcomes

The PEARL registry, a multicenter study of 329 patients with acute DVT treated with PMT, reported technical success in 97% of cases, with complete thrombus clearance in 83% and significant clinical improvement in 94% of patients. The mean procedure time was 2.4 h, with a mean rt-PA dose of 33 mg. Major bleeding occurred in 3.6% of patients, with no procedure-related pulmonary embolism or deaths [75].

The ATTRACT trial, which will be discussed in detail in the clinical outcomes section, included a PMT arm where 92% of patients received AngioJet, Trellis, or another mechanical device in conjunction with thrombolysis [16]. While this trial did not demonstrate a significant reduction in PTS incidence for the overall study population, subgroup analyses suggested potential benefit in patients with iliofemoral DVT [59].

### 4.4. Venous Stenting

Venous stenting plays an important role in the comprehensive management of DVT, particularly for addressing underlying venous stenoses or obstructions that may contribute to thrombus formation and recurrence [74]. Venous stenting may be performed as part of the initial intervention following thrombolysis or thrombectomy, or as a separate procedure for chronic post-thrombotic venous occlusions [83].

#### 4.4.1. Indications and Technical Considerations

The primary indications for venous stenting include the following:

1. Residual venous stenosis after thrombolysis/thrombectomy: Significant stenoses (>50% diameter reduction or pressure gradient >2 mmHg) may be treated with stenting to maintain patency and prevent rethrombosis [74].
2. Extrinsic venous compression: May–Thurner syndrome (compression of the left common iliac vein by the right common iliac artery) is present in up to 50% of patients with left-sided iliofemoral DVT and typically requires stenting for long-term patency [84].
3. Chronic post-thrombotic venous occlusion: In patients with established PTS and venous claudication, recanalization and stenting of chronic occlusions may improve symptoms and quality of life [85].

Venous stenting requires careful consideration of stent type, size, and placement. Dedicated venous stents have been developed to address the unique challenges of the venous system, including high radial strength to resist external compression, flexibility to conform to venous anatomy, and large diameters appropriate for major veins. Examples include the Wallstent (Boston Scientific, Marlborough, MA, USA), Venovo (BD Bard, Franklin Lakes, NJ, USA), Vici (Boston Scientific, Marlborough, MA, USA), and Zilver Vena (Cook Medical, Bloomington, IN, USA) [86].

Stent sizing typically aims for 10–20% oversizing relative to the normal vein diameter [74]. For iliac vein stenting, extension into the inferior vena cava is often necessary to prevent inflow stenosis, while extension below the inguinal ligament is generally avoided when possible [87]. Post-stenting anticoagulation and antiplatelet therapy practices vary, with most centers recommending at least 3–6 months of anticoagulation followed by antiplatelet therapy if anticoagulation is discontinued [16].

#### 4.4.2. Outcomes and Complications

Technical success rates for venous stenting exceed 95% in most series [74]. Primary patency rates range from 74% to 89% at 12 months and 57% to 79% at 24 months, with secondary patency rates of 80% to 94% at 24 months [74,86]. Factors associated with reduced patency include stenting across the inguinal ligament, thrombophilia, and suboptimal inflow or outflow [87].

Complications specific to venous stenting include stent migration, fracture, or compression; in-stent restenosis; and contralateral iliofemoral thrombosis due to venous outflow obstruction [86]. The incidence of these complications varies widely across studies, reflecting differences in patient selection, stent types, and follow-up protocols [74].

### 4.5. Patient Selection for Interventional Procedures

Appropriate patient selection is critical to optimize outcomes and minimize complications of interventional DVT treatment. While specific criteria continue to evolve, several factors have been identified as important considerations:

1. Thrombus location and extent: Interventional approaches are generally reserved for proximal DVT, particularly iliofemoral involvement, where the risk of PTS is highest, and the potential benefit of thrombus removal is greatest [19].
2. Symptom duration: Acute thrombus (<14–21 days) is more amenable to thrombolysis and associated with better outcomes compared to subacute or chronic thrombus [73]. However, successful intervention has been reported in selected patients with symptoms up to 4–6 weeks [88].
3. Bleeding risk: Absolute contraindications to thrombolysis include active internal bleeding, recent cerebrovascular event, intracranial neoplasm, or recent major surgery/trauma. Relative contraindications include recent minor surgery, pregnancy, and uncontrolled hypertension [19]. Pure mechanical approaches may be preferred in patients with elevated bleeding risk [67].
4. Age and comorbidities: Advanced age (>65–75 years) is associated with increased bleeding risk with thrombolytic therapy [62]. Significant comorbidities, limited life expectancy (<1 year), and poor functional status may reduce the long-term benefit of interventional treatment [19].
5. Thrombus characteristics: Fresh, loosely organized thrombus responds better to both thrombolysis and mechanical removal compared to chronic, organized, and wall-adherent thrombus [73].
6. Anatomic considerations: Underlying venous anomalies, stenoses, or compression syndromes may influence the choice of intervention and need for adjunctive stenting [84].
7. The Society of Interventional Radiology and the American Heart Association have published consensus guidelines for patient selection, generally recommending consideration of interventional treatment for patients with acute (<14 days) iliofemoral DVT, severe symptoms, low bleeding risk, good functional status, and life expectancy > 1 year [17,19]. However, these recommendations continue to evolve as new evidence emerges from clinical trials and registries.

## 5. Clinical Outcomes of Interventional DVT Treatment

The evaluation of clinical outcomes following interventional treatment of deep venous thrombosis is essential for determining the role of these procedures in contemporary practice. This section examines the evidence regarding technical success, thrombus removal, venous patency, prevention of post-thrombotic syndrome, quality of life, and safety outcomes across different interventional approaches.

### 5.1. Randomized Controlled Trials

Several randomized controlled trials have compared interventional approaches to conventional anticoagulation for DVT treatment, providing the highest level of evidence available.

#### 5.1.1. The CaVenT Trial

The Catheter-directed Venous Thrombolysis in Acute Iliofemoral Vein Thrombosis (CaVenT) trial was the first randomized controlled trial to evaluate the long-term effects of CDT. This Norwegian multicenter study randomized 209 patients with acute iliofemoral DVT to either conventional anticoagulation alone or CDT plus anticoagulation. The primary outcome was the incidence of PTS at 24 months, assessed using the Villalta scale [21].

At 2 years, the absolute risk reduction for PTS was 14.4% (41.1% in the CDT group vs. 55.6% in the control group, *p* = 0.047), corresponding to a number needed to treat of seven [21]. The reduction in PTS was maintained at 5 years, with an absolute risk reduction of 28% (43% vs. 71%, *p* < 0.001) [58]. Iliofemoral patency at 6 months was significantly higher in the CDT group (65.9% vs. 47.4%, *p* = 0.012). Major bleeding occurred in 3.8% of patients in the CDT group, with no intracranial hemorrhages or deaths [21].

Despite these positive findings, the CaVenT trial had several limitations, including open-label design, use of older thrombolytic protocols without modern mechanical adjuncts, and limited statistical power. Additionally, the control group had a higher-than-expected incidence of PTS, potentially magnifying the treatment effect [16].

#### 5.1.2. The ATTRACT Trial

The Acute Venous Thrombosis: Thrombus Removal with Adjunctive Catheter-Directed Thrombolysis (ATTRACT) trial was a multicenter, randomized, open-label trial that enrolled 692 patients with acute proximal DVT. Patients were randomized to receive either anticoagulation alone or pharmacomechanical catheter-directed thrombolysis (PCDT) plus anticoagulation. The primary outcome was the development of PTS between 6 and 24 months, assessed using the Villalta scale [16].

Contrary to expectations, the trial found no significant difference in the incidence of PTS between the PCDT and control groups (47% vs. 48%, *p* = 0.56). However, moderate-to-severe PTS was reduced in the PCDT group (18% vs. 24%, *p* = 0.04). Major bleeding within 10 days occurred more frequently in the PCDT group (1.7% vs. 0.3%, *p* = 0.049) [16].

Importantly, a pre-specified subgroup analysis of patients with iliofemoral DVT (n = 391) showed a reduction in moderate-to-severe PTS (18.4% vs. 28.2%, *p* = 0.021) and significantly better venous disease-specific quality of life at 24 months in the PCDT group. This suggests that the benefits of interventional treatment may be more pronounced in patients with more proximal thrombus [59].

The ATTRACT trial has been criticized for several methodological issues, including the inclusion of patients with femoropopliteal DVT (who may derive less benefit from intervention), variable use of mechanical thrombectomy devices, and suboptimal stenting practices. Nevertheless, it represents the largest randomized trial of interventional DVT treatment to date and has significantly influenced clinical practice [16].

### 5.2. Other Randomized Trials

The CAVA trial, a Dutch multicenter study, randomized 184 patients with acute iliofemoral DVT to ultrasound-accelerated catheter-directed thrombolysis (UACDT) plus anticoagulation or anticoagulation alone. The primary outcome was PTS at 12 months, assessed using the Villalta scale. The trial found no significant difference in PTS incidence between groups (29% vs. 35%, *p* = 0.42). However, venous patency at 12 months was significantly higher in the UACDT group (95.2% vs. 81.6%, *p* = 0.01) [60].

The TORPEDO trial compared pharmacomechanical thrombolysis to anticoagulation alone in 183 patients with acute proximal DVT. At 6 months, the incidence of PTS was significantly lower in the intervention group (3.4% vs. 27.2%, *p* < 0.001), as was the rate of venous reflux (3.4% vs. 24.5%, *p* < 0.001) [61]. These results are notably more favorable than those of ATTRACT or CAVA, possibly reflecting differences in patient selection, intervention techniques, or outcome assessment [16,60].

### 5.3. Observational Studies and Registries

Numerous observational studies and registries have reported outcomes of interventional DVT treatment, often with larger sample sizes and more diverse patient populations than randomized trials.

The National Venous Registry, one of the earliest large-scale evaluations of CDT, included 287 patients with acute iliofemoral DVT. Complete or partial (>50%) thrombolysis was achieved in 83% of patients, with 1-year patency rates of 60%. Major bleeding occurred in 11% of patients, reflecting the higher thrombolytic doses used in earlier practice [52].

The PEARL registry, focusing on pharmacomechanical thrombectomy, reported technical success in 97% of 329 patients, with complete thrombus clearance in 83%. Freedom from PTS at 6 months was observed in 82% of patients who completed follow-up. Major bleeding occurred in 3.6% of patients, with no procedure-related deaths [75].

The FLASH registry evaluated the safety and effectiveness of the FlowTriever system in 800 patients with acute pulmonary embolism and included a DVT substudy. Among patients with concomitant DVT, technical success was achieved in 95%, with significant improvement in symptoms and minimal complications [79].

For venous stenting, the VIRTUS trial evaluated the Vici venous stent in 170 patients with iliofemoral venous outflow obstruction. Primary patency at 12 months was 84%, with clinical improvement in 79% of patients [89]. Similar results have been reported for other dedicated venous stents, with primary patency rates ranging from 74% to 89% at 12 months [74,86].

### 5.4. Comparative Effectiveness of Interventional Approaches

Few studies have directly compared different interventional approaches for DVT treatment. The available evidence suggests that pharmacomechanical techniques may offer advantages over standard CDT in terms of procedure time, thrombolytic dose, and hospital length of stay [76].

A retrospective comparison of rheolytic thrombectomy versus CDT in 202 patients found similar rates of complete thrombus removal (70% vs. 60%, *p* = 0.14) but significantly shorter procedure times (2.4 vs. 23.4 h, *p* < 0.001) and hospital stays (5.4 vs. 8.9 days, *p* < 0.001) with rheolytic thrombectomy [76].

The PERFECT registry compared outcomes of CDT, pharmacomechanical CDT (PCDT), and PCDT with ultrasound acceleration in 303 patients with acute iliofemoral DVT. Technical success was similar across groups (88% overall), but procedure time and hospital length of stay were significantly shorter with PCDT compared to CDT [62].

More recently, the advent of purpose-designed mechanical thrombectomy devices that require minimal or no thrombolytics (e.g., ClotTriever, FlowTriever) has generated interest in comparing these approaches to traditional pharmacomechanical techniques. The ongoing CLOUT and DEFIANCE trials aim to evaluate the safety and efficacy of the ClotTriever system compared to standard care and may provide valuable comparative data [78].

## 6. Safety Outcomes

Safety concerns, particularly bleeding complications, have been a major focus in the evaluation of interventional DVT treatments.

In the CaVenT trial, major bleeding occurred in 3.8% of patients in the CDT group compared to 1.9% in the control group (*p* = 0.66) [21]. In the ATTRACT trial, major bleeding within 10 days was more frequent with PCDT (1.7% vs. 0.3%, *p* = 0.049), but there was no difference in overall major bleeding during follow-up (12% vs. 8%, *p* = 0.09) [16].

A systematic review and meta-analysis of 19 studies (including 994 patients) reported a pooled major bleeding rate of 7.9% (95% CI 4.7–11.0%) for CDT [54]. More recent studies using lower-dose thrombolytic protocols and modern devices have reported lower bleeding rates, typically 2–5% [16,62].

Other procedure-related complications include access site issues (hematoma, pseudoaneurysm), pulmonary embolism, and device-specific complications such as hemolysis with rheolytic thrombectomy [80]. The incidence of symptomatic pulmonary embolism during interventional treatment is generally low (<1%), though asymptomatic embolization may occur more frequently [16].

Venous stenting carries specific long-term risks, including in-stent restenosis, stent fracture, and stent migration [86]. Rates of clinically significant in-stent restenosis range from 10% to 30% at 3–5 years, often requiring reintervention [74]. Factors associated with increased risk of restenosis include stenting across the inguinal ligament, long segment occlusions, and thrombophilia [87].

## 7. Cost-Effectiveness

The economic impact of interventional DVT treatment must be considered alongside clinical outcomes. The higher initial costs of intervention may be offset by reduced long-term costs associated with PTS management.

A cost-effectiveness analysis based on the CaVenT trial data found that CDT was associated with an incremental cost-effectiveness ratio (ICER) of USD 20,429 per quality-adjusted life year (QALY) gained, which is generally considered cost-effective by conventional standards [90]. A similar analysis using ATTRACT trial data found that PCDT for iliofemoral DVT had an ICER of USD 137,526 per QALY, exceeding typical willingness-to-pay thresholds. However, this analysis was limited by the trial’s 2-year time horizon, which may not capture the full long-term benefits of intervention [91].

A decision analysis model incorporating data from multiple sources estimated that CDT would be cost-effective for patients with iliofemoral DVT if it reduced the absolute risk of PTS by at least 10% [67]. This threshold appears to be met in patients with iliofemoral DVT based on subgroup analyses from the ATTRACT trial and results from the CaVenT trial [21,59].

## 8. Synthesis of Evidence

The available evidence suggests that interventional treatment of DVT, particularly for patients with iliofemoral involvement, can reduce the risk of moderate-to-severe PTS and improve venous patency compared to anticoagulation alone. The magnitude of benefit appears greatest for patients with extensive proximal thrombus, severe symptoms, and treatment within 14 days of symptom onset [16,59].

The conflicting results between trials like CaVenT (positive) and ATTRACT/CAVA (neutral for primary outcome) highlight the importance of patient selection, intervention technique, and outcome assessment in determining the value of interventional approaches [16]. The reduction in moderate-to-severe PTS observed in the iliofemoral subgroup of ATTRACT suggests that focusing interventions on this high-risk population may optimize the benefit-risk ratio [59].

Modern pharmacomechanical approaches appear to offer a favorable balance of efficacy and safety, with shorter procedure times, lower thrombolytic doses, and reduced bleeding risk compared to traditional CDT [76]. Newer purpose-designed mechanical thrombectomy devices may further improve this profile by eliminating or minimizing the need for thrombolytics, though comparative data are limited [78].

## 9. Conclusions

Interventional procedures for deep venous thrombosis have evolved significantly over the past three decades, offering options for active thrombus removal and restoration of venous patency beyond what can be achieved with anticoagulation alone. This systematic review has examined the technical aspects, clinical outcomes, and safety profiles of these approaches, with several key findings emerging from the available evidence.

First, interventional treatment appears most beneficial for patients with acute iliofemoral DVT, where the risk of post-thrombotic syndrome is highest and the potential benefits of thrombus removal are greatest. Subgroup analyses from the ATTRACT trial and results from the CaVenT trial consistently demonstrate greater benefit in this population, particularly for preventing moderate-to-severe PTS. The number needed to treat to prevent one case of PTS in patients with iliofemoral DVT ranges from 7 to 14 across studies, suggesting a clinically meaningful effect.

Second, the evolution of interventional techniques has led to improved safety profiles and procedural efficiency. Modern pharmacomechanical approaches combine the benefits of thrombolysis with mechanical disruption, allowing for more rapid thrombus clearance with lower thrombolytic doses. Newer purpose-designed mechanical thrombectomy devices that require minimal or no thrombolytics may further improve the benefit-risk ratio, though comparative data are still emerging.

Third, appropriate patient selection is critical to optimize outcomes. Factors associated with favorable results include symptom duration less than 14 days, first episode of DVT, extensive thrombus burden (particularly iliofemoral involvement), low bleeding risk, good functional status, and life expectancy greater than one year. Standardized risk assessment tools to guide patient selection are needed and represent an important area for future research.

Fourth, venous stenting plays an important role in addressing underlying venous stenoses or obstructions, which are present in up to 80% of patients with iliofemoral DVT. The development of dedicated venous stents has improved technical outcomes, with primary patency rates of 74–89% at 12 months. However, long-term data on stent durability and optimal antithrombotic management after stenting remain limited.

Despite these advances, several important questions remain unanswered. The optimal timing of intervention, the role of interventional treatment in specific patient subgroups (e.g., cancer-associated thrombosis, recurrent DVT), and the comparative effectiveness of different interventional approaches require further investigation. Additionally, standardization of outcome measures, particularly for PTS assessment, would facilitate more meaningful comparisons across studies.

Future research directions should include the following:

1. Prospective studies focusing specifically on patients with iliofemoral DVT, where the benefit of intervention appears greatest;
2. Head-to-head comparisons of different interventional techniques, particularly newer mechanical thrombectomy devices versus traditional pharmacomechanical approaches;
3. Development and validation of risk prediction models to identify patients most likely to benefit from intervention;
4. Longer-term follow-up studies to assess the durability of treatment effects and late complications;
5. Cost-effectiveness analyses incorporating real-world practice patterns and long-term outcomes.

In conclusion, interventional procedures for DVT offer effective thrombus removal and may reduce post-thrombotic syndrome compared to anticoagulation alone, particularly in patients with iliofemoral DVT. Patient selection remains critical to optimize the benefit–risk ratio. As techniques continue to evolve and evidence accumulates, a more personalized approach to DVT management will likely emerge, with interventional treatment playing an important role in the comprehensive care of selected patients with this common and potentially debilitating condition.

## Data Availability

All data supporting the findings of this study are contained within the article. No additional datasets were generated or analyzed. Any extracted data from included studies can be made available upon reasonable request to the corresponding author.

## Supplementary Materials

The following supporting information can be downloaded at: https://www.mdpi.com/article/10.3390/medicina61081476/s1, Final PRISMA 2020 Checklist (Updated).
