# Supplementary material for: Interventional Procedures in Deep Venous Thrombosis Treatment: A Review of Techniques, Outcomes, and Patient Selection
Source: Medicina (Kaunas). 2025 Aug 18;61(8):1476. doi: 10.3390/medicina61081476 (PMC12388484; doi:10.3390/medicina61081476)
Supplement: Supplementary file 1 [file medicina-61-01476-s001.zip › medicina-3755857-supplementary.pdf]

Final PRISMA 2020 Checklist (Updated)

Section	Checklist Item	Location in Manuscript
Title	Identify the report as a systematic review.	Title page
Abstract	See the PRISMA 2020 for Abstracts checklist.	Structured abstract (PRISMA-compliant)
Rationale	Describe the rationale for the review in the context of existing knowledge.	Introduction, paragraphs 1-2
Objectives	Provide an explicit statement of the objective(s) or question(s) the review addresses.	End of Introduction, paragraph 5
Eligibility criteria	Specify the inclusion and exclusion criteria for the review.	Methods 2.1 Search Strategy and Study Selection
Information sources	Specify all databases, registers, websites, organisations, reference lists and other sources searched or consulted to identify studies. Specify the date when each source was last searched or consulted.	Methods 2.1
Search strategy	Present the full search strategies for all databases, registers and websites, including any filters and limits used.	Methods 2.1 (search terms listed)
Selection process	Specify the methods used to decide whether a study met the inclusion criteria.	Methods 2.1 (reviewers, process)
Data collection process	Specify the methods used to collect data from reports.	Methods 2.2
Data items	List and define all outcomes for which data were sought.	Methods 2.2, 2.3
Data items	List and define all other variables for which data were sought.	Methods 2.2, 2.3
Study risk of bias assessment	Specify the methods used to assess risk of bias in the included studies.	Results 5.X (summary of risk of bias)
Effect measures	Specify for each outcome the effect measure(s) used.	Methods 2.3
Synthesis methods	Describe the methods of synthesis used.	Methods 2.4

Reporting bias assessment	Describe any methods used to assess risk of bias due to missing results.	Methods 2.4
Certainty assessment	Describe any methods used to assess certainty (or confidence) in the body of evidence.	Results/Discussion (certainty of evidence discussed)
Study selection	Describe the results of the search and selection process.	Methods 2.1; PRISMA flow diagram
Study characteristics	Cite each included study and present its characteristics.	Results sections 4–5
Risk of bias in studies	Present assessments of risk of bias for each included study.	Not reported in full
Results of individual studies	Present for all outcomes, for each study: summary statistics and effect estimates.	Results section 5
Results of syntheses	Summarize the results of all statistical syntheses conducted.	Results section 5.4; 6
Reporting biases	Present assessments of risk of bias due to missing results.	Methodology section
Certainty of evidence	Present assessments of certainty (or confidence) in the body of evidence.	Results/Discussion (certainty of evidence discussed)
Discussion	Provide a general interpretation of the results in the context of other evidence.	Discussion section
Registration and protocol	Provide registration information for the review.	Methods 2.5 (registration statement)
Support	Describe sources of financial or non-financial support.	Funding section
Competing interests	Declare any competing interests.	Conflict of Interest section
Availability of data	Report which materials are publicly available.	Availability of data section
